# Impact of *Drosophila* Models in the Study and Treatment of Friedreich’s Ataxia

**DOI:** 10.3390/ijms19071989

**Published:** 2018-07-07

**Authors:** Véronique Monnier, Jose Vicente Llorens, Juan Antonio Navarro

**Affiliations:** 1 Unité de Biologie Fonctionnelle et Adaptative (BFA), Sorbonne Paris Cité, Université Paris Diderot, UMR8251 CNRS, 75013 Paris, France; 2 Department of Genetics, University of Valencia, Campus of Burjassot, 96100 Valencia, Spain; 3 Lehrstuhl für Entwicklungsbiologie, Universität Regensburg, 93040 Regensburg, Germany

**Keywords:** *Drosophila melanogaster*, Friedreich’s ataxia, frataxin, iron, oxidative stress, metal homeostasis, lipid metabolism, endoplasmic reticulum, genetic screens, drug screens

## Abstract

*Drosophila melanogaster* has been for over a century the model of choice of several neurobiologists to decipher the formation and development of the nervous system as well as to mirror the pathophysiological conditions of many human neurodegenerative diseases. The rare disease Friedreich’s ataxia (FRDA) is not an exception. Since the isolation of the responsible gene more than two decades ago, the analysis of the fly orthologue has proven to be an excellent avenue to understand the development and progression of the disease, to unravel pivotal mechanisms underpinning the pathology and to identify genes and molecules that might well be either disease biomarkers or promising targets for therapeutic interventions. In this review, we aim to summarize the collection of findings provided by the *Drosophila* models but also to go one step beyond and propose the implications of these discoveries for the study and cure of this disorder. We will present the physiological, cellular and molecular phenotypes described in the fly, highlighting those that have given insight into the pathology and we will show how the ability of *Drosophila* to perform genetic and pharmacological screens has provided valuable information that is not easily within reach of other cellular or mammalian models.

## 1. Molecular and Cellular Aspects of Friedreich’s Ataxia

### 1.1. Pathophysiology of the Disease

Friedreich’s ataxia (FRDA) is an autosomal recessive degenerative disease present only in Indo-European and Afro-Asiatic populations [1]. It is the most frequent autosomal recessive ataxia in the Caucasian population. The major clinical features of FRDA include age of onset around puberty, progressive ataxia, muscle weakness, sensory loss and non-neurological features such as skeletal defects and cardiomyopathy. FRDA neuropathology starts with the degeneration of the large sensory neurons of the dorsal root ganglia (DRG), followed by atrophy of the dorsal columns that produces loss of proprioception and vibration sense. Degeneration of the spinocerebellar tracts of the spinal cord results in upper motor weakness. Atrophy of the dentate nuclei is also observed and accounts for the cerebellar component of ataxia. The neuronal degeneration is also accompanied with demyelination of sural nerves and DRG fibres. Most FRDA patients develop hypertrophic cardiomyopathy with thickened septum walls and iron deposits in the myocardium [2,3]. Other clinical signs are diabetes mellitus and carbohydrate intolerance [3] and in agreement, postmortem analysis of pancreas from FRDA patients revealed loss of β cells in islets of Langerhans [4]. Although the expression of frataxin is ubiquitous [5,6], the selective tissue vulnerability and cell death in FRDA is still far from understood.

Reduced expression of the nuclear-encoded protein frataxin is the molecular cause of the disease. Such a reduced expression originates from the presence of a guanine-adenine-adenine (GAA) expansion in the first intron of the gene [5]. However, around 2–4% of FRDA patients are compound heterozygotes with a GAA expansion on one allele and a small mutation on the second allele [7]. The GAA has been shown to alter chromatin structure and to induce epigenetic modifications that lead to a reduced transcription of the gene [8], whereas the point mutations induce a loss-of-function [7]. Human frataxin (FXN) is translated in the cytoplasm as a precursor of 210 amino acids that is imported into the mitochondria [6]. It is then proteolytically cleaved by the mitochondrial processing peptidase (MPP) in a two-step process that leads to the successive generation of an intermediate form of 19 kDa and of the mature form of 14 kDa [9,10]. Frataxin is a protein highly conserved among eukaryotes and some prokaryotes. Sequence alignment of the frataxin family shows two distinct regions, an N-terminal block of 70–90 amino acids that is absent in prokaryotes and poorly conserved among eukaryotes, and a highly conserved block of 100–120 residues in the C-terminus of the protein [11,12].

The three-dimensional structure of the human frataxin, the *Escherichia coli* homolog (*CyaY*) and the Yeast frataxin homolog 1 (*Yfh1*), have been fully characterized [13,14,15]. All three proteins share conserved C-terminal regions that consist of an antiparallel β-sheet flanked by two α-helices. The N-terminal tail present in eukaryotes appears to be intrinsically unfolded. The presence of acidic residues within the first α-helix and the edge of the first β strand forms a negatively charged surface that is involved in iron binding, whereas a neutral flat area on the β-sheet probably allows protein-protein interactions [12,16]. A crucial difference among frataxin orthologs is their ability to undergo iron-dependent oligomerization. Moreover, the functional relevance of such oligomers is still controversial. In the presence of excess of iron, Yfh1 has been shown to assemble into trimmers, hexamers and to larger 12-mers, 24-mers and 48-mers [17,18]. However, some authors suggest that such oligomers are dispensable since a yeast frataxin defective for oligomerization was able to perfectly replace the endogenous protein [19]. Unlike CyaY and Yfh1, the mature form of the human frataxin did not seem to form oligomers [20], although recent experiments suggest the opposite [21].

The function of frataxin has been linked to different mitochondrial pathways, but still remains partially unclear. The most accepted hypothesis based on all the available data supports the participation of frataxin in iron-sulfur (Fe–S) cluster biosynthesis in the mitochondrial matrix [22]. Fe–S clusters constitute one of the most ancient and ubiquitous of the biological prosthetic groups. More than 200 types of proteins, exhibiting a remarkable functional and structural diversity, contain Fe–S centres. Fe–S clusters are composed by two or more iron atoms bridged by sulfide centres, most frequently in a [2Fe–2S] or a [4Fe–4S] conformation [23]. In higher eukaryotes, Fe–S biogenesis takes place in the mitochondria by means of homologous components of the bacterial Fe–S system that were transferred from the bacterial endosymbiotic ancestor of this organelle. Mitochondrial de novo Fe–S cluster biogenesis occurs in two steps. The first step involves the assembly of inorganic iron and sulfur on a scaffold protein, IscU in bacteria and Isu1 in yeast. It is known that this reaction needs a cysteine desulphurase as sulfur donor, IscS in bacteria and Nfs-1-Isd11 in yeast, whereas the origin of the iron still needs to be fully elucidated. In the second step, the clusters are transferred from the scaffold to recipient apoproteins for incorporation within specific amino acid residues [23].

The involvement of frataxin in this biosynthetic process was first suggested by the deficient activity of proteins containing Fe–S clusters in FRDA patients and in mouse models [24,25]. Additional data supporting this hypothesis was provided by studies in *Saccharomyces cerevisiae* [26]. Analysis using mammalian recombinant proteins further characterized the interaction of human frataxin with a preformed complex composed of NFS1, ISCU and ISD11 [20,27]. However, the exact role of frataxin in the Fe–S cluster assembly process is still a matter of debate. On one hand, the iron-binding properties of the human protein indicated that frataxin acts as the iron donor in the first step of the assembly reaction [28]. On the other hand, other groups showed that frataxin functions as an allosteric regulator that facilitates the transfer of sulfur from NFS1 for the assembly and that iron is not required for the interaction between human frataxin and the NFS1/ISCU/ISD11complex [20,27,29]. Surprisingly, CyaY seems to inhibit the Fe–S cluster biogenesis [30]. In addition to its primary role in Fe–S cluster biogenesis, frataxin has been proposed to act directly as a chaperone that provides iron to aconitase [31] and ferrochelatase [32] or electrons to ubiquinone/mitochondrial respiratory chain complex II [33] via protein-protein interaction.

### 1.2. Current and Prospective Treatments

Several clinical trials have been or are currently conducted to evaluate pharmacological compounds in FRDA patients. These trials are based on various strategies, such as increasing frataxin expression, lowering oxidative stress, improving mitochondrial function, reducing iron-mediated toxicity or modulating frataxin-controlled metabolic pathways [34]. However, to date, there is no pharmacological treatment with demonstrated efficacy to cure or even to stop the progression of the disease. It is therefore crucial to identify new candidate molecules for pharmacological approaches, and we believe that *Drosophila* models have a major role to play in this process.

An alternative, complementary and promising future approach is the possibility of a gene therapy. Two preclinical studies, performed on cardiac and sensory mouse models of FRDA using adeno-associated virus (AAV) vectors to express frataxin, showed that intravenous injection of an AAV vector expressing FXN not only prevented the development of cardiac and neurological features, but also improved cellular functions when injections were made in symptomatic mice [35,36]. The major challenges in the development of such gene therapy approaches will undoubtedly be to target efficiently the affected cells and tissues in FRDA patients and to succeed in inducing frataxin expression at levels that remain physiological. Indeed, studies in *Drosophila* showed that overexpression of endogenous or human frataxin in the nervous system decreased longevity, affected locomotor activity, and induced neurodegeneration [37,38], revealing the importance of a finely controlled level of expression of frataxin in the development of gene therapy approaches. Very recently, a study using human cells confirmed the hypothesis from these fly reports [39].

## 2. Analysis of Frataxin Function in *Drosophila melanogaster*

*Frataxin homolog* (*fh*) is the *Drosophila* homolog of *FXN*. The gene *fh*, of 965 bp, is in the region 8C/D of the X chromosome. It is composed of one intron flanked by two exons, encoding for the 190 amino acid *Drosophila* frataxin protein (Fh). Frataxin is a highly conserved protein, and the *Drosophila* protein shares conservation in both sequence and structure. Preliminary in silico analysis predicted the presence of a signal peptide for mitochondrial import [40]. The mitochondrial localization of fly frataxin was corroborated by a co-localization experiments in cell culture [41]. The biophysical and iron binding properties of the *Drosophila* frataxin are consistent with those from orthologs. Interestingly, Fh is stable as an iron-repleted monomer, not very prone to form oligomers and delivers iron to the scaffold on Fe–S cluster assembly [42]. This might be an advantage to study frataxin function. Furthermore, there has been some controversy regarding the existence and the function of an extramitochondrial frataxin pool that seemed sufficient to enhance survival [43] or to revert iron deregulation in FRDA cells [44]. *Drosophila* offers a nice experimental scenario to assess this issue in vivo. In agreement with evidence from *Trypanosoma brucei* [45], expression of a fly frataxin lacking the mitochondrial signal peptide failed to rescue the defects induced by the mutation present in the *fh*^1^ allele compared to the full length frataxin [46].

### 2.1. Methodologies Applied to Generate FRDA Models in Flies

The fruit fly is a versatile model organism used in biomedical research to study a broad range of phenomena. The use of disease models is essential to understand the pathophysiological mechanisms of human disorders. For over a century, different approaches have been developed to obtain *Drosophila* models that recapitulate the hallmarks of human pathologies. Many of them take advantage of the GAL4/UAS system. This bipartite system, adapted from yeast, involves two transgenes and allows, up to some degree, the spatial and temporal control of the expression of the transgene of interest [47]. On the one hand, one that carries the GAL4 transcription factor under the control of a given promoter and on the other hand, the construct of interest downstream of an upstream activating sequence (UAS). Using this approach, the fly has successfully mimicked human conditions by overexpressing mutant forms of human genes [48,49] or by promoting posttranscriptional silencing by means of the RNA interference (RNAi) strategy [50], of your favorite gene [51].

Using the RNAi strategy, John P. Phillip’s lab developed the first *Drosophila* model of FRDA [52]. This work was the first showing that loss of frataxin in flies reproduced the main molecular and biochemical features of the human disorder. Therefore, it was pivotal to stablish *Drosophila* as an excellent platform to reveal factors that can modulate the phenotypes and mechanisms involved in the disease. This first RNAi construct consisted of two inverted repeated copies of the first 391 nucleotides of *fh* cloned into the pUAST vector. Authors generated three transgenic lines—UDIR1, UDIR2 and UDIR3 (also named as fhRNAi, DfhIR, fhRNAi-1 in the literature)—able to almost completely suppress the expression of FH protein [52,53]. These lines have been also combined with the gene switch system [54,55] at the lab of Hervé Tricoire [56]. In this system, a modified GAL4 protein is fused to a progesterone steroid receptor, allowing the regulation of its GAL4 activity via the presence or absence of the synthetic progesterone analogue mifepristone (RU486). Using the same methodology and a different construct, Maria Dolores Moltó’s group developed a second RNAi model [41] named UAS-*fh*IR (also known as fhRNAi-2). In this model, two copies of the *fh* cDNA were cloned in the pUAST vector in an opposite direction and separated with a fragment of the GFP sequence as a linker to facilitate the formation of the loop [57]. Although based in a similar idea, there is a critical difference between both models. As stated above, the UDIR lines strongly abolish frataxin expression whereas, the UAS-*fh*IR line triggers a moderate and mild reduction of frataxin levels (around 70% compared to controls) down to levels that better resemble the frataxin expression found in FRDA patients [58]. Importantly, this line has the advantage to allow working with adult flies upon ubiquitous downregulation of frataxin expression [41,59,60,61], whereas the UDIR lines are extremely useful to unveil pathological events and mechanisms upon tissue-specific silencing [56,61,62,63,64]. In this review, we decided to keep the original names—UDIR and UAS-*fh*IR—throughout the entire manuscript.

The last model was recently developed in Hugo Bellen’s lab. In an outstanding effort to unravel genes of the X chromosome involved in neuronal function and likely in neurodegeneration [65], authors performed an ethyl methanesulfonate (EMS) based mosaic genetic screen of lethal mutations. Using this mutagenic alkylating agent, they identified a missense mutant allele of *fh* (S136R, named *fh*^1^) [46]. The mutation is located in a highly conserved region of the protein used for the binding of frataxin to the ISC machinery [66]. Such a change triggers a strong loss of function leading to developmental arrest in larval stages. Remarkably, the fly stock used in the EMS screen also allowed the authors to carry out a mosaic mutant analysis by generating mitotic clones of adult photoreceptor neurons using the eyeless flippase and flippase recognition target (FLP/FRT) system [67]. This way, it is possible to generate a fly mutant for frataxin in a non-vital organ/tissue, while the rest of the fly remains wild-type like.

These are the models of FRDA developed so far in *Drosophila melanogaster*. All phenotypes induced by these genetic tools/models were recently described in detail [68]. Therefore, in the next chapters, we will focus on the detailed analysis of how all these models have contributed to the understanding of the pathophysiological mechanisms of the disease and the implications of such discoveries.

### 2.2. Fly Models Recapitulate FRDA Features

RNAi-mediated frataxin ubiquitous inactivation, using the *daughterless* (*da*-GAL4) driver, leads to developmental lethality. Third instar-larvae (L3) fail to pupariate or reach the pupal stage much later than controls and are not capable of becoming viable adults [52]. Similarly, *fh^1^* hemizygote mutants are lethal at the L3 or pupal stages, with prolonged larval stages. Removal of the maternal source of wild-type frataxin in these mutants leads to an earlier lethality, occurring at the embryonic stage [46]. This shows that frataxin is an essential protein during early embryogenesis in *Drosophila*, similarly to observations on mouse frataxin knock-out mutants [69] and in line with the lack of FRDA patients containing point mutations in both frataxin alleles [70].

To study the effects of partial frataxin inactivation on adults, and thus more closely mimic the situation of human FRDA patients, several strategies were used to bypass the preadult lethality. First, taking advantage of the fact that the activity of the UAS-GAL4 system is temperature dependent, some *da*-GAL4>UDIR adults were obtained by switching flies from 25 to 18 °C at the beginning of the pupal stage. These adults are mostly short-lived, with a peak of mortality between 3 and 6 days of adult life. Interestingly, the cohort of flies still alive after this initial high-mortality phase could live up to 40 days of age, suggesting that frataxin is particularly needed in the early days of adulthood [52]. Another study used the *actin*-GAL4 ubiquitous driver to express the UAS-*fh*IR construct that allowed experiments on viable adults due a moderate but significant reduction of frataxin expression, as indicated above. Such flies also exhibited decreased lifespan and defective climbing activities [41].

In addition to these systemic approaches, a major advantage of RNAi-based *Drosophila* models lies in the ability to target specific tissues and therefore to evaluate which tissue or organs are particularly sensitive to frataxin depletion and to study tissue-specific physiopathological mechanisms.

Several studies targeted frataxin inactivation in neurons. Surprisingly, frataxin inactivation using various neuronal drivers resulted in viable progeny. Noticeably, flies in which RNAi-mediated frataxin inactivation is induced in the larval brain (*c698a*-GAL4) or in specific subtypes of neurons such as motorneurons (*D42*-GAL4 driver) or dopaminergic neurons (*Ddc*-GAL4) are viable without any obvious phenotypes [41,52]. Reduced adult lifespan and climbing activity were observed using the *neur*-GAL4 and *C96*-GAL4 drivers, allowing frataxin inactivation in the peripheral nervous system, suggesting that these neurons might be more sensitive to frataxin deficiency [41]. In agreement, in our unpublished observations, we did not observe any significant effect on fly fitness when frataxin was downregulated in all neurons by means of *Elav*-GAL4 and a moderate effect when using the RU486 inducible *Elav*-GS driver (as described in [71]). Silencing of frataxin in glial cells, using the pan-glial *Repo*-GAL4 driver, leads to partial developmental lethality, reduced adult lifespan and impaired locomotor activity [63]. Following this initial discovery in *Drosophila*, studies on human and mouse astrocytes confirmed a detrimental effect of frataxin silencing in astrocytes with non-cell autonomous effects on neurons, showing that glial cells are highly susceptible to contribute to the FRDA disease [72,73].

Heart-specific developmental inactivation of frataxin in flies, using the RU486 inducible Hand-GS driver, leads to cardiac dilatation and impaired systolic function [56]. Remarkably, those alterations are very similar to cardiac dysfunctions observed in patients and mouse models of FRDA [25,74,75]. Importantly, these phenotypes are fully rescued by complementation with human frataxin, showing conserved cardiac functions of frataxin between *Drosophila* and mammals. Interestingly, adult-specific frataxin inactivation did not lead to cardiac phenotypes, showing that the fly heart is particularly sensitive to frataxin depletion during developmental stages before adulthood. In human FRDA patients, the cardiomyopathy can already be observed in children, and patients with an earlier onset of disease generally also showed more severe cardiac involvement, suggesting a similar specific requirement of cardiac frataxin in young humans before adulthood [75]. It would be interesting to know whether such a developmental component is crucial exclusively in the heart or whether it is also a key element in other tissues/organs of the fly. Analyzing this aspect is of high interest in light of the new inducible and reversible mouse model [76] in which reactivation of frataxin expression after 12 weeks is sufficient to stop the progression of the disease and to recover morphological features in the affected heart and DRG.

Frataxin downregulation in muscles using the *Mef2*-GAL4 driver leads to reduced longevity and locomotion, associated with expected mitochondrial dysfunctions [62]. Although often considered as secondary in the disease, pathological manifestations are also observed in muscles of FRDA patients. Those defects include loss of muscle strength, particularly affecting the lower limbs and prolonged recovery of calf muscle following exercise [77,78]. Moreover, magnetic resonance spectroscopy of the calf muscles in FRDA patients has also demonstrated impairment of ATP synthesis and inadequate oxygen utilization [78,79,80]. Therefore, *Drosophila* can be considered as an attractive and pertinent organism to study muscular dysfunctions, besides heart dysfunctions described above, induced by frataxin deficiency.

Finally, inactivation of frataxin in the steroidogenic prothoracic gland (the gland that produces ecdysteroids involved in larval molts, pupariation and metamorphosis) was sufficient to lead to developmental lethality. Although this appears at first glance to be specific to insect physiology, it could eventually reveal cellular and metabolic dysfunctions induced by frataxin deficiency relevant in the context of the FRDA disease [81].

As summarized in Table 1, ubiquitous and tissue-specific inactivation of frataxin allowed recapitulation of clinical features characteristic of the FRDA disease, such as reduced lifespan, impaired locomotor activity, cellular degeneration and cardiac dysfunction, providing relevant models to study the physiopathological mechanisms involved in the disease at various scales, to screen for genetic and pharmacological modifiers and ultimately propose therapeutic strategies.

## 3. The Contribution of *Drosophila* to the Analysis and Cure of FRDA

### 3.1. Understanding the Molecular Physiopathology of the Disease

As shown in Section 2, frataxin depletion in the fly strongly resembles the physiological effects observed in FRDA patients. Starting from this premise, the labs of John P. Phillips, Maria Dolores Moltó, Stephan Schneuwly, Peter J Hollenbeck, Hervé Tricoire, and Hugo Bellen investigated the molecular and cellular pathological mechanisms underlying the defects described in fly fitness upon frataxin downregulation. Those labs exploited the possibility to easily perform in fly genetic screens because of the outstanding available genetic toolbox. Moreover, *Drosophila* has shown in several instances its pivotal ability to react to pharmaceuticals compounds, similar to humans [82,83]. Such an ability facilitates testing of several hundreds of drugs in a short period of time [84,85]. Both strategies have allowed the unveiling of novel molecules and mechanisms that seem to be crucial for the etiology of the disease.

#### 3.1.1. Biogenesis of Fe–S Clusters: The Beginning of the End

**Figure 1:**
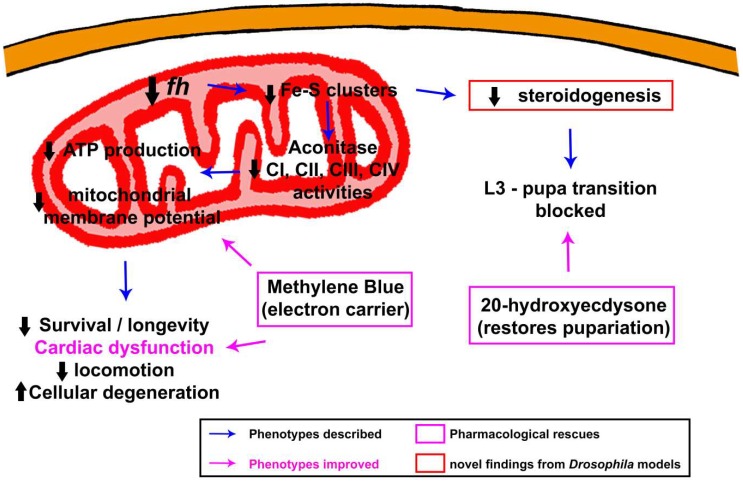
Graphic summary of molecular defects and rescues described in *Drosophila melanogaster* models of frataxin-deficiency in relation to Fe–S clusters generation and mitochondrial function.

As mentioned previously, frataxin deficiency leads to a deficit in Fe–S cluster biogenesis in many organisms, including yeast, plants and mammals. Fe–S clusters are essential cofactors of proteins located in mitochondria, cytosol and nucleus, essential for many cellular processes such as respiration, replication, DNA repair, ribosome biogenesis and iron regulation. Importantly, the cellular machinery involved in Fe–S synthesis is conserved between mammals and flies [86]. Thus, frataxin inactivation is susceptible to affect many cellular and physiological functions that are pivotal during development and aging. In agreement, activities of several mitochondrial Fe–S proteins have been shown to be affected by frataxin deficiency in *Drosophila* and are presumably responsible for subsequent mitochondrial dysfunctions (Figure 1). Strong ubiquitous RNAi-mediated frataxin inactivation dramatically reduced activities of mitochondrial aconitase and of respiratory chain (MRC) complexes I, II, III and IV [52], whereas moderate reduction only provokes a reduction of aconitase activity and complexes I, II and indirectly complexes III and IV were normal [41]. Interestingly, human frataxin expression restores the aconitase activity in frataxin-deficient flies, further showing that these two proteins share similar functions in both organisms [38]. Reduced activity of MRC complex I, associated with an increased ADP/ATP ratio, was also observed in the *fh^1^* mutant [46]; frataxin knockdown in muscles leads to impaired aconitase activity, ATP production and mitochondrial membrane potential [62].

Ubiquitous or specific inactivation of frataxin in the steroidogenic prothoracic gland leads to a hormonal deficit in the steroid hormone 20-hydroxyecdysone, responsible for the developmental blockage at the third instar larval stage and the formation of giant larvae described previously. These hormonal defects are also likely to be a consequence of defective Fe–S cluster assembly, leading to decreased activities of Fe–S enzymes involved in steroidogenesis such as ferredoxins or *Neverland* (*Nvd*), which convert cholesterol into 7-dehydrocholesterol. Interestingly, frataxin silencing decreases progesterone synthesis in human KGN ovarian granulosa cells, suggesting that the involvement of frataxin in steroid synthesis is a conserved function of frataxin protein from flies to human and that steroidogenesis could be affected in FRDA patients [81].

Although loss of Fe–S containing enzymes is a common feature of all FRDA fly models, it is striking to observe that some unexpected differences are also present. The differences between UDIR and *fh*IR models are likely related to levels of frataxin downregulation achieved with both. The comparison suggests that aconitase is much more sensitive to a reduction of Fe–S generation than the MRC complexes as it has been seen in FRDA patients [24]. However, it is difficult to understand that the flies containing a mutant allele of frataxin triggering a severe loss-of-function only display a clear reduction in Complex I activity, but not of other mitochondrial respiratory chain complexes.

#### 3.1.2. Alterations in Iron Metabolism: The Cornerstone behind FRDA

Keeping in mind the toxic biochemistry of iron surplus, the first aspect to study downstream of the impairment of Fe–S cluster formation in frataxin deficient cells is the fate of the mitochondrial iron that cannot be incorporated into the clusters. The first answer came around 40 years ago from biopsy studies from patients [87,88] in which mitochondrial iron deposits were detected in cardiac tissue. However, such accumulations are not a general feature and, for example, in DRG or DN, iron redistribution and not iron accumulation seems to be the main consequence [89,90]. Unexpectedly, results from patients’ samples revealed a high variability in the total iron content that prevent concluding that there are significant changes in the total amount of this transition metal [89,90,91,92].

Under these circumstances, *Drosophila* might represent a nice approach because of the genetic and phenotypical homogeneity of this model organism. Indeed, when these questions were addressed in the fly models, the results were robust and clear. Importantly, frataxin deficiency in flies triggers a strong accumulation of mitochondrial iron [46,93]. However, levels of total iron inversely correlate with amounts of functional frataxin. On one hand, total iron remained unaltered upon partial reduction of *fh* [93], whereas a clear increase was detected in flies with a drastic loss-of-frataxin function [46]. Remarkably, *Drosophila* has been the first FRDA model organism showing that iron also accumulates in the nervous system [46]. In this report, Chen and collaborators also suggested that lack of detection of ferric iron in the nervous system of mouse models [25,69,94,95] was likely a methodological issue rather than absence of iron accumulation. In a second manuscript, the same group showed that implementation of Fe^3+^ detection is sufficient to visualize iron accumulation in the nervous system of a new FRDA mouse model [96]. It would be interesting and of high relevance to apply this new iron-detection method to all fly and mouse FRDA models in order to definitively clarify this issue. In agreement with the presence of a detrimental mitochondrial iron accumulation, frataxin deficiency triggers hypersensitivity towards iron supplementation in food. Increased iron content further impaired pupariation [46,52] and worsened the reduced longevity [59] or the neuronal activity [46] of frataxin-deficient individuals. This is in agreement with cell culture models [97,98], although a diet enriched with iron was able to ameliorate the heart dysfunction of the muscle creatine kinase (MCK) conditional knockout mouse model [99].

**Figure 2:**
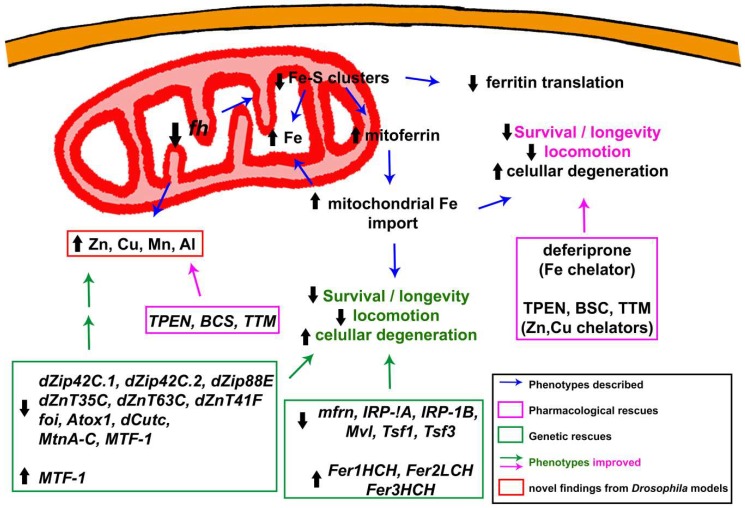
Graphic summary of molecular defects and rescues described in *Drosophila melanogaster* models of frataxin-deficiency in relation to iron metabolism and homeostasis of other metals.

As indicated above, an outstanding advantage of *Drosophila* over other model organisms is the possibility to modulate the frataxin knockdown using different RNAi constructs. In this sense, a fly model with moderate reduction of frataxin will allow identification of primary events compared to the broader panoply of changes triggered by complete loss of frataxin function. With this strategy, Navarro and colleagues were able to provide an insight into the molecular mechanisms behind iron toxicity. Importantly, as nicely reviewed in [100,101], the main actors of iron metabolism are conserved between humans and flies. This ensures the translation of fly results into clinical outcomes. Authors found changes in three genes: (i) Mitoferrin, the main mitochondrial iron importer; (ii) the iron regulatory protein 1A (*IRP-1A*) also known as the cytosolic aconitase, which is involved in the translation of other iron-related genes depending on the iron availability via Iron Responsive Elements (IREs) at the 5′UTR and 3′UTR of the transcripts, and (iii) ferritin, the key protein required for iron storage in *Drosophila*. In short, frataxin silencing upregulated mitoferrin along with a strong cytosolic scarcity that impaired ferritin translation because of an increase expression of *IRP-1A*. These results are in agreement with those reported in the cardiac tissue of the MCK frataxin knock-out mice [25]. This group of phenotypes clearly suggests different genetic strategies to improve FRDA conditions in fly models. Downregulation of mitoferrin and thus, reducing mitochondrial iron transport, was sufficient to recover the cellular iron metabolism to a control-like situation [59]. This concomitantly rescued aconitase activity as well as the brain degeneration observed upon frataxin downregulation in glia. Unexpectedly, coexpression of ferritins failed to recover those phenotypes in these RNAi-based models [59]. On the other hand, promotion of iron chelation by overexpressing ferritin subunits or mitochondrial ferritin successfully suppressed neuronal degeneration in fly photoreceptors [46] and increased longevity of UAS-*fh*IR flies [59]. Such a difference between models might be attributed to differences in the iron dyshomeostasis, which seems to be more severe in the *fh*^1^ mutant allele [46]. Soriano and colleagues followed a similar strategy in a new biased genetic screen. In this case, authors impaired cellular iron uptake by knocking down the expression of *Malvolio* (*Mvl*), *Tsf1* and *Tsf3* (the *Drosophila* homologues of the mammalian Divalent metal transporter-1 and of iron Transferrins, respectively). Silencing of all three genes improved locomotion as well as eye morphology in frataxin deficient flies. They also tested the effect of *IRP-1A* and *IRP-1B* silencing to counteract the upregulation of *IRP-1A* detected previously [59]. As expected, suppression of IRP activity elicits a positive effect on FRDA phenotypes. Although no rescue mechanism was reported in this case, the reduction of mitochondrial iron accumulation driven by IRP1 depletion in mouse livers mutants for frataxin suggests a possible explanation [102]. All these results are summarized in Figure 2. As suggested above, *Drosophila* models display a reduced iron-storage capacity in the cytosol due to a decrease in ferritin protein levels [52,59]. The same defect has been found in mouse and worm FRDA models [103,104]. In contrast to all three animal models, histological analyses of patient’s samples suggest cytosolic iron accumulation instead of iron depletion in DRG, DN, satellites cells and heart [89,90,105,106]. Although the reasons of these discrepancies still remain unclear, such a remarkable difference might be attributed to the specific biology and iron homeostasis in each model, tissue, or cell type. For example, ferritin accumulates in the liver of a mouse FRDA model, whereas it decreases in the heart of the same model [99].

It has been largely speculated that a mitochondrial signal might act as an iron sensor regulating the activity of IRPs and, in turn, iron metabolism. Such a signal would not be present in frataxin deficient cells, leading to the loss of iron equilibrium. We can speculate that the Fe–S clusters themselves might be the missing link. Recently, Fanis Missirlis suggested that mitochondrial superoxide dismutase (SOD2) might be the key molecule. This is interesting since Fe can replace Mn in the SOD2 catalytic core, leading to inactivation of the enzyme. This will boost oxidative stress, generating a feedback loop that further contributes to inactivation of aconitase [86]. In agreement, genetic reduction of SOD1 and SOD2 in flies affects aconitase activity [107]. This hypothesis fits with the reduced SOD2 activity displayed in the yeast FRDA model [108]. However, SOD2 activity seems to not be decreased in FRDA flies [52], suggesting that other signals might be participating in the process. Deciphering the precise nature of this signal would facilitate the complete understanding of iron dyshomeostasis in FRDA.

The last contribution of the fly in unraveling the toxic mechanism of iron in FRDA was reported a couple of years ago by Hugo Bellen’s lab [46]. Authors describe a pathological mechanism that includes sphingolipid synthesis and Pdk1/Mef2 signaling downstream of iron accumulation. This mechanism will be further discussed later.

#### 3.1.3. To Be or Not to Be: The Controversy of Oxidative Stress

Oxidative stress can be defined as an imbalance between the production of Reactive Oxygen Species (ROS) and the antioxidant systems that protect the cell from them. Mitochondria are the main source of ROS. The consequences of increased ROS production or reduction of ROS protection include damage in DNA and RNA by incorporating oxidized bases or oxidizing bases already integrated in the DNA [109], in proteins by oxidizing backbone and amino-acids [110] and in lipids by the formation of lipid peroxyl radicals and hydroperoxides that can damage many cellular structures [111].

As stated above, alterations caused by frataxin deficiency include impaired Fe–S cluster biogenesis with the concomitant aconitase and respiratory chain dysfunctions and mitochondrial iron accumulation [112]. Those defects have been proposed to alter the cellular redox status and lead to oxidative damage in FRDA. This is a hallmark of FRDA that may further contribute to the progression of the disease [113]. Three mechanisms have been suggested to participate: (i) the enhanced ROS production due to the uncoupling of the Electron Transport Chain (ETC) [24]; (ii) the increased ROS production through the Fenton chemistry due to an accumulation of free iron [114] and (iii) the impairment of antioxidant response [115]. Furthermore, since ROS also act as signaling molecules in the nervous system [116], their dysregulation may well disturb other cellular processes in addition to non-specific oxidative damage.

The relation of oxidative stress and FRDA in all models has been recently nicely summarized by Annalisa Pastore’s group [117]. In short, two main manifestations of such redox alterations are present in the FRDA literature. On one hand, loss of frataxin function triggers the production of oxidative stress biomarkers in yeast [6,108,118,119,120,121,122], *C. elegans* [123], mouse [25,95] as well as in FRDA patients and patients-derived samples [97,115,122,124,125,126,127,128,129]. On the other hand, frataxin deficient cells have been shown to display increased sensitivity towards antioxidant insult [97,98]. However, those markers are absent in other FRDA models [76,130] or in different brain regions from FRDA patients [131].

Although treatments based on antioxidants were promising in preclinical studies, they show a very limited benefit in patient clinical trials [132,133]. Thus, it is crucial to understand the true influence of oxidative stress in the pathophysiology of FRDA. We can only speculate about the inefficacy of this type of drugs in clinical trials. It is possible that the moment of application of the treatment is crucial. In some preclinical models, the compound could possibly be administered in the early stages of the disease, in which case improvement is feasible. However, in Phase III (last phase) clinical trials, the heterogeneity of patients, including some displaying more severe symptoms, avoids a clear analysis of these chemicals.

**Figure 3:**
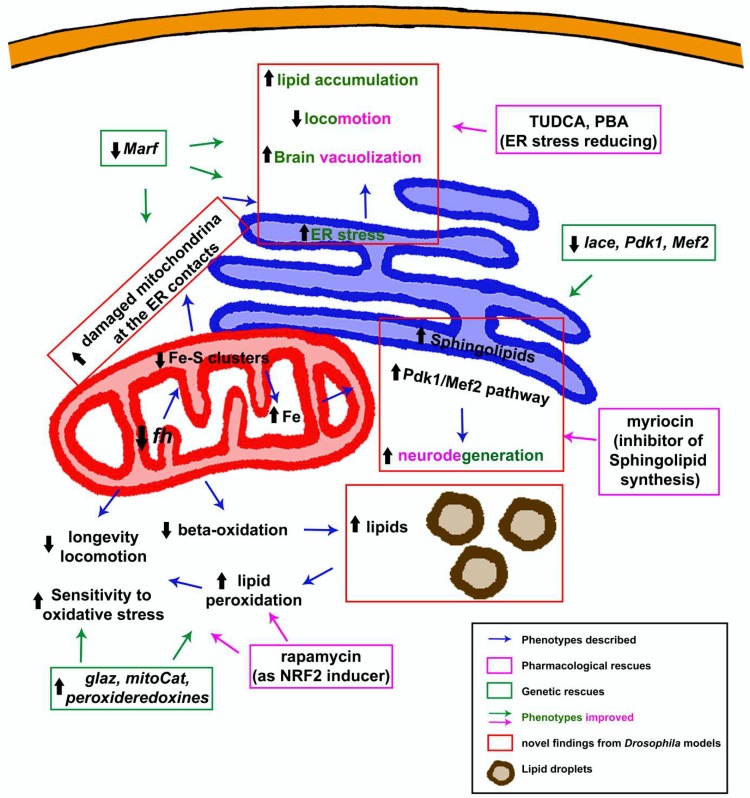
Graphic summary of molecular defects and rescues described in *Drosophila melanogaster* models of frataxin-deficiency in relation to oxidative stress and lipid metabolism.

To this purpose, researchers have analyzed three parameters in fly models: (i) increased production of ROS; (ii) enhanced sensitivity towards oxidative insults and (iii) positive effect of antioxidant treatments (Figure 3). Unfortunately, contradictory results have been obtained. On one hand, frataxin deficiency in glia, muscle and ubiquitously induced hypersensitivity to oxidative stress [41,62,63] and accumulation of lipid peroxides [60,63]. On the other hand, increased ROS levels have not been reported when frataxin was silenced in larval motor neurons [64] or in *fh^1^* flies that carry a missense mutation in the *fh* locus that triggers a strong loss-of-function [46]. This was unexpected since these mutant flies exhibit a clear complex I deficiency that is normally associated with increased ROS production [134,135,136]. Moreover, because drugs based on combating oxidative stress are being used to treat FRDA patients [132,133], *Drosophila* studies have tried to recover FRDA phenotypes by overexpressing antioxidant enzymes. Initially, the suppression of defects in the PNS was achieved by coexpression of some scavenging enzymes such as catalase, mitochondrial catalase and mitochondrial peroxiredoxin [53]. Moreover, the lack of recovery by both cytosolic and mitochondrial superoxide dismutase (SOD1 and SOD2, respectively) [52] strongly suggested that hydrogen peroxide (H_2_O_2_) and not superoxide (O_2_^−^) free radicals were key contributors to the pathology. Similar results were obtained when antioxidant defenses were activated in glial cells in which frataxin was silenced. Indeed, only hydrogen peroxide scavengers were able to improve the negative geotaxis impairment [62]. The hypothesis is in line with observations in a FRDA mouse model in which overexpression of SOD1 and a SOD2 mimetic also failed to improve FRDA conditions [130]. As previously mentioned, Fe might replace Mn in the catalytic site of SOD2 and thus, in a situation of mitochondrial iron accumulation such as FRDA, overexpression of SOD2 will not guarantee an increase activity of this enzyme [86]. Peroxiredoxins are one of the hydroperoxides scavengers that showed a positive impact on PNS of frataxin-deficient flies [53]. Although they have not been further used in the fly models, their interplay with aging signaling pathways such as p38 MAPKinase [137] and the recent identification of this kinase as a therapeutic agent in FRDA [138] might indicate that further studies with peroxiredoxins are of great interest.

However, overexpression of catalase or treatment with a synthetic catalase mimetic did not improve heart function in the impressive FRDA fly heart model [56] and failed to suppress the neurodegeneration observed in photoreceptors of *fh^1^* mutant flies [46]. This might indicate a lack of contribution of ROS in the neurological and cardiac phenotypes associated with FRDA. We can also guess that the relevance of specific ROS subtypes might be a tissue-specific effect. It is difficult to extract definitive conclusions when every parameter is not studied in each model. For example, it would be interesting to know whether oxidative stress enhances the cardiac dysfunction and neurodegenerative phenotypes observed in FRDA fly models or if the presence of lipid peroxides is a common feature of all FRDA models. We have studied redox imbalance in two situations in which frataxin deficiency triggered hypersensitivity to oxidative insult [62,63] by means of a redox sensitive GFP, named roGFP, which changes its excitation maximum from 488 to 405 nm when oxidized [139]. In particular, we used a fusion of the glutaredoxin (Grx1) to a mitochondrial targeted roGFP, which allows effective measurement of the ratio of oxidized glutathione (GSSG) to reduced glutathione (GSH) within this organelle [140]. Glutathione is a powerful cellular antioxidant capable of neutralizing reactive oxygen species and becomes oxidized after donating an electron [141]. In our unpublished observations, we observed higher levels of reduced glutathione in frataxin-deficient glial and muscle cells. This result is in contrast to findings in the erythrocytes of a cohort of FRDA patients [125], in skeletal mouse muscles and motorneurons [142,143], in yeast [120,122] and in human cell cultures [122,144] in which total GSH levels were low and the ratio of GSSG to GSH was increased. However, such ratio is not altered in neurons of the FRDA mouse model [145]. One possible explanation for the lack of oxidized glutathione in flies is that the GSSG/GSH ratio was measured at time points where mitochondria are strongly affected and thus less ROS are produced as a byproduct of respiration. This might also partially explain the contradictory results regarding the involvement of oxidative stress. Up to this moment, the only evidence regarding glutathione in fly models was an increase of total glutathione observed upon ubiquitous downregulation of frataxin [60] without additional information of the contribution of each form.

Finally, in line with the idea that oxidative stress might play a role in the pathology, it was found that a slight overexpression of fly frataxin increases resistance to hydrogen peroxide [146], whereas a strong overexpression makes the flies more sensitive [37,38].

#### 3.1.4. Beyond Iron: Importance of Other Metals in the Development and Progression of the Pathology

As explained exhaustively above, there are many studies that provide evidence for the role of frataxin in iron homeostasis, in both humans and model organisms. Interestingly, few studies on biopsy samples of Dentate Nucleus (DN) and DRG from FRDA patients have shown loss of homeostasis of other metals [147,148,149]. Those studies describe the redistribution of other crucial transition metals such as copper (Cu) and zinc (Zn) and the expression of proteins involved in metal-trafficking such as metallothioneins. However, no further mechanism was offered in those reports. Because of this, the group of María Dolores Moltó started a pioneer research that aimed to better understand the molecular basis underlying the altered homeostasis of these metals and to reveal whether those alterations are able to suggest alternative pathways for therapy development (Figure 2). As reported by Soriano and colleagues [61], ubiquitous frataxin deficiency led to a significant accumulation of aluminium (Al), manganese (Mn), Zn and Cu. In addition, it seems that frataxin-deficient flies are more vulnerable to Al. The authors suggested that Al exposure resulted in iron dyshomeostasis and ROS [150]. In line with these results in the fly, it was recently shown that yeast frataxin is able to bind Cu and Mn, even with higher affinity than Fe. Concomitantly, frataxin-deficient yeast display enhance sensitivity towards exogenous Cu and Mn and accumulate Cu in mitochondria, albeit to a lesser extent compared to iron [151]. In agreement, the role of frataxin in Cu metabolism has been also suggested in plants [152]. Taking these data into account, a biased genetic screen was carried out with key regulators of Zn and Cu homeostasis such as Zn transporters (Zips and ZnTs) and Cu chaperones. This is important since beneficial effects triggered by Zn and Cu genes have never been addressed in any FRDA model. Authors observed that downregulation of genes involved in Cu and Zn regulation improved FRDA conditions in the fly [61]. For example, silencing of *Drosophila Atox1* orthologue recovered locomotion and eye degeneration. Loss of function of *Atox1* induces Cu accumulation in intestinal cells and prevents Cu transport into other cell types [153]. This might abolish the accumulation of Cu in critical tissues in FRDA flies. It is easy to speculate whether frataxin may be acting as a mitochondrial Cu chaperone in the given circumstances, as has been suggested for Fe [31]. In case of Zn, downregulation of genes involved in the transport of Zn into (*Zip* genes) and out of (*ZnT* genes) the cytoplasm rescued FRDA phenotypes. Although it is initially difficult to reconcile the results from groups of genes, it is possible that altering one of them is sufficient to influence the rest of the genetic network controlling Zn homeostasis, leading to the same positive result. In addition, some of the *Zip*/*ZnT* genes also seem to be able to transport Fe [154,155]. This property might also contribute to reducing mitochondrial iron accumulation in a manner that has not been studied yet. Supporting those results, the overexpression of MTF-1 (Metal-responsive Transcription Factor-1), the master regulator of gene expression under metal stress conditions, suppresses locomotor phenotypes in a FRDA *Drosophila* model [61].

#### 3.1.5. Dyshomeostasis of Lipid Metabolism: Much More Than a Supporting Actor

Some years ago, Navarro and collaborators found that downregulation of frataxin in glia cells triggered a massive accumulation of lipid droplets [63]. The first histological evidence was further corroborated by means of gas chromatography coupled with mass spectrometry (GC/MS). This lipidomic approach showed that only a subgroup of free fatty acids (FFA) was really contributing to the accumulation observed. Such analysis identified myristic acid (C14:0), palmitic acid (C16:0), palmitoleic acid (C16:1), oleic acid (C18:1) and linoleic acid (C18:2) as most abundant FFA in frataxin-deficient cells. Although lipid droplets were previously described in cardiac muscles of a FRDA mouse model [25], this *Drosophila* study was the first that considered them as a pathological element. Indeed, presence of lipid droplets have been associated with neurodegeneration in other fly models [156,157]. Following this logic and considering that lipids are extremely prone to oxidation by Fenton’s reaction, authors also found increased levels of lipid peroxides in FRDA flies. Reduction of these free radicals by co-expression of the scavenger *glial lazarillo* was sufficient to improve some FRDA phenotypes [63] (Figure 3). Recently, reduction of lipid peroxides in fibroblasts and neurons from different FRDA mouse models have also improved FRDA phenotypes [158,159]. Although Navarro and coworkers suggested that the boost of lipoperoxides and not the lipid accumulation was the driving force behind the phenotypes, this issue was not completely clarified. Moreover, the authors hypothesized that the impairment of FFA beta-oxidation because of loss of mitochondrial function would lead to their accumulation. This hypothesis is supported by the interaction in yeast between frataxin and the electron transfer flavoprotein complex (ETF), which transfers electrons from beta-oxidation into ubiquinone [33]. However, transcriptomic studies in mouse models and patient samples [160,161] suggested that other pathways might also be participating in the derangement of lipid homeostasis. For example, downregulation of the peroxisome proliferator-activated receptor gamma (PPARγ) expression or an increased lipogenesis via upregulation of the sterol-responsive element-binding protein 1 (SREBP1) have been reported in FRDA. Furthermore, in absence of aconitase activity (the most affected enzyme in FRDA), citrate accumulates and is exported to the cytosol where it promotes lipid biogenesis [86]. Therefore, it is worthwhile to further analyze these pathways in fly FRDA models as well as others that are related to lipid metabolism in humans, and flies such as Adenosine monophosphate-dependent protein kinase (AMPK) [162].

Although the lipid accumulation found in the FRDA flies seems to differ from the pathological features found in *postmortem* analysis of FRDA patients [163], several indications suggest lipid dyshomeostasis in the patients [129,164,165,166,167,168]. Since this fly report from Navarro et al. [63], the accumulation of lipids has been reported in mouse, rat and human cell culture FRDA models [169,170,171] and remodeling of lipid metabolism has been observed in *C. elegans* [172]. In addition, FFA seem to further reduce frataxin expression [171]. Interestingly, a lipotoxic-induced cardiomyopathy has already been described in the fly [173] and it is characterized by similar phenotypes to those reported by Tricoire and collaborators in their cardiac fly FRDA model [56]. Moreover, accumulation of lipids has also been found after silencing of *dCPT2* or *ND23* in *Drosophila* glial cells [157,174], suggesting that this might be a common defect of mitochondrial dysfunction in glia. On the other hand, no lipid accumulation or just minor defects were observed when genes were knockdown in neurons. Similarly, frataxin silencing in neurons also failed to induce lipid accumulation (Navarro, unpublished observation).

It is important to mention that frataxin inactivation in fly photoreceptor neurons also triggers accumulation of lipids in glia [46]. The same effect has been shown in photoreceptor neurons that are mutant for other mitochondrial genes [175,176]. All this indicates the existence of a specific mechanism coupling photoreceptor neurons and their accessory glia. A recent report suggests that in response to an increase in oxidative stress, glia fuel neurons with lactate. This promotes lipid biogenesis in neurons that are transferred back to the glia, producing the lipid droplets [176]. Whether the same mechanism is occurring in *fh^1^* mutant photoreceptors stills need to be elucidated, since no increased oxidative stress was found in this case [46]. 

As indicated above, lack of frataxin activity triggers accumulation of iron in the fly’s nervous system [46]. It is known than in yeast, sphingolipid synthesis mediates iron toxicity [177]. Therefore, Chen and collaborators investigated the role of this pathway in their fly model. Interestingly, authors concluded that, in agreement with the yeast study, biogenesis of sphingolipids was also increased in frataxin mutant flies. Remarkably, the excess of these lipids, in turn, activated the expression of 3-phosphoinositide dependent protein kinase-1 (*Pdk1*) and myocyte enhancer factor-2 (*Mef2*), which resulted in degeneration and loss of photoreceptors in the ommatidia of mutant frataxin flies. In line with the proposed mechanism, silencing of serine palmitoyltransferase (lace), the limiting factor in the generation of sphingolipid, as well as of *Pdk1* and *Mef2,* partially reverted the cellular degeneration (Figure 3). At this point, it was of extreme relevance to establish whether this pathological mechanism was exclusive to fly models or whether it was conserved among different models. Analysis of a new FRDA mouse model based on a CRISPR/Cas9 strategy as well as heart samples from FRDA patients also revealed activation of the *Pdk1*-*Mef2* pathway downstream of accumulation of iron [96]. This suggests a conserved mechanism and highlights, once more, the relevance of *Drosophila* models in the study of FRDA. However, the mechanism might not be as universal as it was initially thought, and other factors might contribute. The *Pdk1*-*Mef2* pathway is not consistently active in a new reversible mouse model [76], although it displays the characteristic iron deposits. It is also reasonable to speculate that the sphingolipids might be triggering deleterious effects by alternative mechanisms. Dihydrosphingosine (dhSph), dihydroceramide (dhCer), ceramide (Cer) and sphingosine (Sph) levels are increased in *fh^1^* flies and such sphingolipids have been already associated with cellular degeneration in different fly tissues [156,173,178,179,180,181]. For a comprehensive and detailed review about frataxin and lipid metabolism, please check the recent manuscript from Tamarit and collaborators [182].

#### 3.1.6. The Endoplasmic Reticulum Factor: An Alternative Vision to the Pathology

A recent fly report indicates, for the first time, that Endoplasmic Reticulum (ER) stress plays critical roles in the development and progression of frataxin loss-of-function phenotypes in glia [62]. The presence of ER stress markers or hypersensitivity to ER stress has already been reported in FRDA models. Different cell culture models (neuroblastoma, human embryonic kidney cells, rat pancreatic cells, human lymphoblasts among others) and knock-out cardiomyocytes from the MCK mouse model display increased levels of the activating transcription factor (ATF4), the chaperone binding immunoglobulin protein (BiP) and phosphorylated α subunit of eukaryotic translation initiation factor 2 (eIF2α) [4,183,184,185,186]. Edenharter and collaborators identified *Drosophila* Mitofusin (*Marf*) as a suppressor of frataxin-deficient phenotypes in glia, including brain degeneration and lipid dyshomeostasis (Figure 3). Comprehensive analysis of this genetic interaction pointed towards modulation of ER stress as the pivotal element underlying the rescue mediated by *Marf* knockdown. The results presented in this manuscript showed that the roles of *Marf* in the mitochondrial fusion or as a substrate of Pink1-Parkin pathway were not important contributors. Furthermore, authors detected increased levels of ER stress markers in all FRDA models tested, including *fh^1^* mutant flies [62]. Furthermore, it would also be of enormous importance to further characterize the effects downstream of ER stress. For example, some results in flies show that ER stress promotes the activation of the transcription factor ATF4 and such an event is able to induce metabolic remodeling, altering insulin responsiveness and upregulating glycolysis [187,188,189]. Moreover, increased levels of phosphorylated eI2Fα have also been related to dendritic loss upon mitochondrial dysfunction in the fly [190]. Are such responses also activated in frataxin-deficient flies and in other FRDA models? Are they responsible for the metabolic alterations observed in FRDA patient samples [168] and contributing to the axonal “dying back” phenotype?

Interestingly, there is a growing body of findings that highlights the relevance of ER stress in several human neurodegenerative diseases [191]. In this sense, ER stress is also a central element in the pathological mechanism of fly models of other neurological disorders such as Parkinson’s [192], Alzheimer’s [193] and Gaucher’s [194,195] diseases.

The relevance of ER stress in FRDA is also supported by the detection of elevated ER stress levels even prior to deficits in Fe–S biogenesis [184] and by the rescue of phenotypes reducing ER stress without improving mitochondrial function [62]. However, the causes that place ER stress at the core of the FRDA pathology are still obscure. Different possibilities can be inferred from the existing literature. On one hand, it seems that the upregulation of ER stress markers follows the depletion of ER calcium stores in a FRDA cell model [186]. Calcium-handling defects have been observed in several models of FRDA and modulation of calcium signaling is able to ameliorate FRDA defects [196,197,198,199]. However, this aspect has not been addressed yet in any fly FRDA work. Such an analysis would be feasible, since the main elements in the regulation of cellular calcium metabolism are conserved in flies [200,201,202]. In addition, it has been already shown that, in flies, mitochondria are required for calcium homeostasis and physiological calcium levels are essential for mitochondrial transport in neurons [203]. On the other hand, it has been suggested by experts in iron biology that in flies, iron might be mainly stored bound to ferritin in the ER [86]. Interestingly, sphingolipids are synthesized in the ER [178] and, both iron and sphingolipids are known to mediate neurodegeneration in FRDA flies [46]. Remarkably, the mitochondria-associated membranes (MAMs) are cellular structures that connect mitochondria and ER, and both organelles exchange several substrates such as calcium or sphingolipids. Moreover, MAMs have raised a hub of neurodegeneration in several human diseases [204]. Analysis of MAMs in FRDA fly models is compulsory in order to validate this hypothesis and establish a pathological mechanism that links the ER and the mitochondria more precisely.

#### 3.1.7. To Be Continued: Additional Pathways That Might Be Participating

In this section, we would like to present a couple of interesting and promising pathological /therapeutical pathways that we, and others, have published or observed in fly FRDA models, but are not completely exploited.

**Figure 4:**
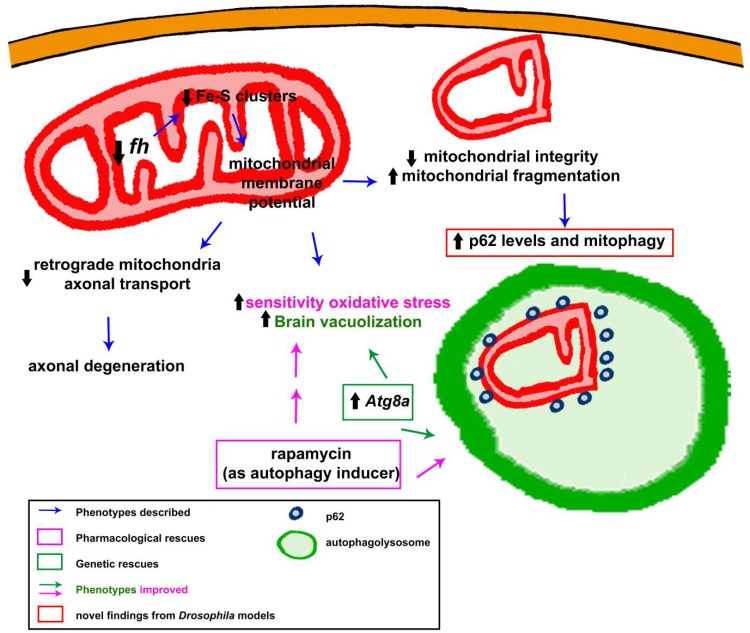
Graphic summary of molecular defects and rescues described in *Drosophila melanogaster* models of frataxin-deficiency in relation to the cellular mitochondrial network.

Although frataxin is involved in the bioenergetics of mitochondria, little is known regarding the influence of frataxin deficiency on mitochondrial dynamics, transport and degradation in a frataxin-deficient context. However, changes in mitochondrial morphology have been reported in several models, including the fly [46,62]. Edenharter and collaborators performed on the fly a molecular analysis of the mitochondrial fusion and fission pathway [62]. The authors analyzed the expression levels of the main regulators of mitochondrial fusion (*Opa1* and *Marf*), mitochondrial fission (*Drp1*), mitochondrial biogenesis (*tfam* and *spargel*) and mitochondrial quality control (*Pink1* and *parkin*). Interestingly, authors observed in the fly musculature increased expression of *Opa1* and reduction of *Drp1* expression, indicating promotion of mitochondrial fusion. Authors suggested that this was a pathological consequence of frataxin deficiency. However, further experiments are needed to better understand this phenotype. A similar analysis was carried out in the yeast FRDA model a few years ago that did not detect significant differences [205]. The fruit fly is the first organism examined to study the efficiency of mitochondrial transport in frataxin-deficient neurons [64]. Shidara and Hollenbeck effectively describe diminished anterograde and retrograde transport in the distal axons of larval motorneurons, which might account for the dying back neuropathy [64]. This result was corroborated much later in a mouse model [198]. Analysis of mitochondrial morphology in the *Drosophila* indirect flight muscles revealed a strong mitochondrial fragmentation. These structural changes normally precede the degradation of those organelles. In agreement, the autophagy marker p62 was dramatically increased and it formed vesicle-like structures, engulfing damaged mitochondria [62]. These results agree with those reported in mouse models of the disease in which autophagy structures or markers tend to accumulate [94,95,185]. However, this result may also raise the question whether autophagy is impaired in FRDA flies or not, as it is normally indicated by p62 accumulation [206]. Further experiments have unambiguously proven that autophagy is enhanced and not affected in FRDA flies [62]. Such an induction of basal autophagy has also been found in *C. elegans* [103,172] and in cell culture [186]. All these results have been summarized in Figure 4.

Finally, during the analysis of the iron metabolism in frataxin-deficient flies, we also detected changes in the expression of the master regulators of hypoxia response. This is in agreement with data from the MCK (heart conditional) mouse model [104]. The hypoxia signaling is conserved in *Drosophila*, which is driven by the two-fly hypoxia inducible factors (HIFs)—*sima* (HIFα ortholog) and *tango* (HIFβ ortholog) [207]. As this happens in higher organisms, they are also stable under hypoxia in the fly but degraded under elevated oxygen conditions [207]. We have found that upon ubiquitous silencing of frataxin, *sima* and its partner *tango* were significantly upregulated (our unpublished observations). Such an upregulation may suggest that frataxin-deficient cells also sense low oxygen conditions. However, we cannot rule out alternative explanations. For example, mouse frataxin is regulated in a HIF-dependent manner due to the presence of a Hypoxia Responsive Element (HRE) at position-1947 in its promoter [208]. Interestingly, the promoter sequence of the fly frataxin also contains a HRE sequence (ACGTG). In agreement, *sima* downregulation lowers the expression of *fh* (data not shown). Altogether, these facts might well suggest that *sima* upregulation is a cellular compensatory response by enhancing frataxin expression. Therefore, in line with current strategies that stimulate frataxin expression to treat patients [209], HIFs might be an interesting possibility. Indeed, such HIFs-frataxin axis seems to be cardio protective against myocardial infarction [210]. In addition, since several reports indicate that hypoxia changes mitochondrial biology in the fly [211,212,213], we can suggest that this is a pathway worth studying in depth in the FRDA context. Remarkably, a recent paper from Schiavi and collaborators has added a new level of complexity to the relation between hypoxia and FRDA [103]. In this manuscript, authors describe that the prolonged longevity of their *C. elegans* frataxin-deficiency model is mediated by an iron-starvation response that activates HIF, among others, leading to enhanced mitophagy. Since, cytosolic iron scarcity is also seen in fly [59] as well as in mouse [104] FRDA models, we can speculate that this might be a compensatory mechanism conserved throughout evolution.

### 3.2. Revealing Possible Therapies in FRDA Fly Models

#### 3.2.1. Analysis of Specific Drugs

Based on the molecular defects and mechanisms shown above in Section 3.1, researchers have also tried to improve FRDA conditions in *Drosophila* by means of pharmacological treatments.

María Dolores Moltó’s lab performed the first chemical treatments as a proof of concept to validate *Drosophila* models as an avenue to identify therapeutic molecules. In this first attempt, Soriano and colleagues aimed to improve mitochondrial function using idebenone. Idebenone is a synthetic analogue of Coenzyme Q10, with the ability to enhance mitochondrial respiration by improving the electron flux along the electron transport chain and also acting as a free-radical scavenger [214]. Idebenone treatment increases lifespan and climbing activities of flies ubiquitously depleted for frataxin, using the *actin*-GAL4 driver as well as longevity of flies with frataxin depletion specifically in the peripheral nervous system (PNS) with *neur*-GAL4 driver [93]. However, idebenone failed to prevent cardiac dilatation or defective systolic function of Drosophila frataxin-deficient hearts, suggesting that the efficacy of idebenone treatment is dependent on affected tissues [56]. It should be noted that clinical trials have not yet been able to show a clear effect of idebenone on the progression of the disease [215]. Methylene Blue, another compound with electron carrier properties that bypass mitochondrial complexes I-III has also been evaluated on the *Drosophila* cardiac model (Figure 1) and fully prevented cardiac dysfunctions [56]. The potential of this type of drug to improve mitochondrial function has been very recently confirmed in patient’s lymphocytes [216]. This compound is thus a promising candidate, but it has not yet been evaluated on murine models or in clinical trials.

Although fly models display discrepancies regarding the detection of ROS markers and the rescue ability of antioxidant enzymes, the effect of antioxidant treatments has been also analyzed. Calap-Quintana and colleagues observed that rapamycin treatment reduced levels of lipid peroxides in the UAS-*fh*IR model [60] and this was sufficient to restore some additional FRDA phenotypes. Importantly, the authors showed that rapamycin drove its effects by promoting protection against oxidative stress via the Cnc-Keap1 pathway (Figure 3). Cap-n-collar (Cnc) is the fly orthologue of mammalian NRF2. NRF2 is the master regulator of the expression of several antioxidant genes such as SOD1 and 2 [217]. Interestingly, activation of NRF2 is impaired in frataxin-deficient cells [115,145,218]. Importantly and in agreement with these *Drosophila* results [60], chemical induction of NRF2 expression is neuroprotective in FRDA motorneurons [219]. Unfortunately, rapamycin failed to improve the effects of strong frataxin depletion in glia [62]. Furthermore, rapamycin is known to induce autophagy in flies [220]. Edenharter and collaborators already showed that mitochondrial clearance by mitophagy (a special form of autophagy) was enhanced in frataxin-deficient glia and muscles [62]. In addition, genetic stimulation of autophagy by overexpression of Atg8a (Figure 4) reduced brain degeneration in a FRDA fly model [62]. Therefore, drugs promoting autophagy might be beneficial. Such approach was tested a few years ago in cell culture [221] and yeast [222] with some level of success in reducing cell death and ROS production, respectively. However, experiments in higher organisms were still required. *Drosophila* is the first multicellular FRDA model that has shown that promotion of autophagy with rapamycin [60] was sufficient to improve fly survival under oxidative stress insult and to recuperate aconitase activity. Since rapamycin does not affect frataxin expression in flies [60], speeding up the degradation of damaged mitochondria will concomitantly reduce oxidative stress and other downstream defects, and improve fly fitness (as summarized in Figure 4). This hypothesis has been nicely demonstrated by Marobbio and coworkers [222]. Although, it is reasonable to consider that effects of the drug over antioxidant enzymes and autophagy are independent, a recent publication on *Drosophila* links the regulation of p62 and Atg8 to Cnc [223].

Besides the genetic interactions between frataxin and iron-related genes, pharmacological manipulation of iron metabolism has also presented beneficial effects in FRDA fly models. Two different molecules—deferiprone (DFP) [93] and desferrioxamine B (DFOB) [224]—were active in vivo and improved longevity and fly locomotion (Figure 2) and transition from larvae to pupa, respectively. While DFP was shown to remove the excess of mitochondrial iron and to prevent Fenton’s reaction, DFOB seems to avoid iron precipitation in the mitochondria increasing, in turn, its bioavailability. An additional group of compounds displaying thioamide function was suggested to act through the chelation of iron, but further experiments are required to prove this capacity [224]. Unfortunately, clinical trials using iron chelators as therapy reported contradictory results ranging from improvement to inefficacy and even further deterioration [91,225,226,227,228]. It has been shown that iron chelators are able to negatively affect frataxin expression as well as activity of mitochondrial enzymes [229,230]. In agreement, mitoferrin knockdown in flies [59] or IRP1 depletion in mouse [102] also worsened some other phenotypes in the respective FRDA models. All this might suggest that precise and accurate control of iron amount is crucial for the efficacy of these types of therapies. The cellular iron concentration might vary among patients and thus, personalized treatment is necessary. Since one of the most important effects of accumulation of toxic iron seems to be the increased generation of sphingolipids, chemical inhibition of sphingolipid biosynthesis has also been evaluated. Myriocin, a drug that suppresses serine palmitoyltransferase function (Figure 3), has also improved neurodegeneration in frataxin-deficient fly photoreceptors [46]. Unfortunately, there is not enough data on the benefits of a therapy based on attacking this pathway in higher organisms.

Due to the accumulation of other metals in a fly model [61], it was interesting to test whether their chelation was also able to counteract some effects triggered by frataxin loss-of-function (Figure 2). Remarkably, fly food supplementation with Zn and Cu chelators (BCS, Bathocuproine disulphonate; TPEN, *N*,*N*,*N*′,*N*′-tetrakis(2-pyridinylmethyl)-1,2-ethanediamine and TTM, Tetrathiomolybdate), also improved the locomotor ability of the FRDA flies [61]. It is known that Cu might interfere with Fe–S cluster production, further aggravating this defect in FRDA flies [86]. This might explain the positive effect of Cu chelation seen by Soriano and colleagues. Therefore, it would be interesting to test whether this new therapeutic approach is able to improve other *Drosophila* loss-of-frataxin phenotypes in glia, muscle, heart and photoreceptor neurons as well as in other cellular and mouse models. Finally, the impact of ER stress [62] was further corroborated due to the protection conferred by two drugs that reduce ER stress (Figure 3). Tauroursodeoxycholic acid (TUDCA) and 4-phenylbutyric acid (PBA) successfully reduced ER stress, cellular degeneration and partially restored aconitase activity in frataxin-deficient flies [62]. It would be important to assess whether increased levels of ER stress markers are present in other fly tissues and if TUDCA or PBA also exert a positive effect on longevity.

All this should be considered as a solid proof of concept to use the fly as an outstanding model to test drugs that might counteract FRDA conditions.

#### 3.2.2. Unbiased Drug Screens

To date, two pharmacological screenings have been performed on fly models of FRDA. The first one was not strictly unbiased. Instead, *Drosophila* models were used for secondary screening of compounds identified in yeast [224]. More than 6,000 compounds issued from the French National Chemical library and the Prestwick Chemical library were first tested for their ability to improve the growth of frataxin-deficient yeast cells. From this primary screening, 12 compounds were selected and then further evaluated in *Drosophila* for their ability to improve the capability of larvae ubiquitously depleted for frataxin to reach the pupal stage. Significant improvement was observed for six of them. One compound, LPS 01-04-L-G10 is particularly interesting, since it also allows reduction in heart dilatation in the cardiac model. Its mechanism of action remains to be elucidated. This study illustrates the interest of coupling complementary models for unbiased approaches to identify new molecules with protective effects. The second is an unbiased pharmacological screening recently performed on the *Drosophila* cardiac model of FRDA [231]. In this study, 1280 compounds of the Prestwick chemical library were tested in vivo for their ability to prevent cardiac dysfunction. Eleven drugs were significantly protective. The drug with the strongest effect was paclitaxel, a microtubule-stabilizing drug. This, together with the observation that the microtubule network was fully disrupted in frataxin-deficient cardiomyocytes, suggests that cardiac dysfunction results at least in part from alterations of the microtubule network.

## 4. Future Perspectives

We have shown that the fly has been a pioneer, unveiling novel pathological mechanisms that will contribute to better understanding of the complete puzzle that the etiology of the disease seems to be, as well as identifying new potential treatments. We hope that these works will inspire researchers on vertebrate FRDA models to investigate the impact of other metals, sphingolipids metabolism, ER stress, and the microtubule network on the pathophysiology of the disease and analyze the attenuation of such defects as therapy in patients.

The panoply of possibilities that the fruit fly offers to inactivate frataxin and the constantly growing genetic toolbox of *Drosophila* place this organism in a privileged position to be a leader model organism in the analysis of frataxin function. We are convinced that the fly will continue providing valuable information by means of: (i) generation of new FRDA models, (ii) characterization of new phenotypes or disease biomarkers and, (iii) development of pharmacological and genetic screens.

(i) Current RNAi-based FRDA models have the major advantage to better mimic frataxin levels in patients, compared to models based on frataxin’s complete loss of function. However, a weakness of these RNAi models is that they are based on the generation of long double strand RNA fragments [41,52]. Because of this, these models display two major limitations. On one hand, the long hairpins are more prone to generating off-target effects [232,233]. On the other hand, Dicer2 in *Drosophila* neurons shows weak expression [234]. This protein plays a key role in the processing of long dsRNA into small (21–23 nucleotides) functional molecules. This would explain the weak (or none) effects observed when the current RNAi models were applied to silence frataxin in the fly CNS [41,52], as summarized in Table 1. Accordingly, Shidhara and Hollenbeck only detected around 30% reduction of frataxin expression in motor neurons with UDIR2 line [64], when the same RNAi line decreased frataxin expression to undetectable levels using a ubiquitous GAL4 driver. Indeed, coexpression of Dicer2 in neurons dramatically increased frataxin downregulation effects in neurons (data not shown). Therefore, it would be highly interesting to downregulate frataxin by means of new RNAi lines based on a short hairpin strategy (as described by Norbert Perrimon’s lab [235]) that bypasses the need of coexpressing Dicer2 and excludes the presence of off-targets.

Although the recently developed approach of genome editing by clustered, regularly interspaced, short palindromic repeat (CRISPR) technology [236] has been successfully applied in the fly in the last years [237] even to model diseases such as hereditary spastic paraplegia [238], there are no reports in the FRDA field. Interestingly, Simon Bullock’s lab recently combined the UAS/GAL4 system with the CRISPR/Cas9 strategy; this will allow to easily generate neurons-specific frataxin mutants flies in a reasonable short period of time [239]. The CRISPR/Cas9 tools will also facilitate the generation of FRDA fly models based on introduction of a GAA repeat expansions in the *Drosophila* frataxin gene intron. As indicated above, the presence of a pathological GAA expansion in the first intron of the human gene is the most common alteration in the human frataxin locus in FRDA patients [5]. This will provide an outstanding platform to test drugs and pathways that might help revert the effect of the expansion by interfering with GAA-induced silencing mechanisms or to evaluate genome-editing strategies to delete the expansions.

(ii) Although FRDA patients show clear peripheral neuropathy [2] and fly and human sensory neurons are strongly similar [240], little has been done to analyze the integrity and neurodegenerative mechanisms in adult *Drosophila* PNS. Interestingly, alterations in sphingolipid metabolism, similar to those described in frataxin deficient photoreceptors [46], are also participating in sensorimotor neuropathy in a fly model of hereditary sensory and autonomic neuropathy type 1 (HSAN1) [241]. The approach, recently implemented at the lab of Stefan Thor to use leg neurons as a model system, is an appealing possibility [242]. Moreover, recent studies have started to reveal cognitive defects in FRDA patients, such as deficits in memory and social skills that have remained unexplored for a long time [243]. The analysis of the behavior of frataxin-deficient flies is mostly limited to longevity and locomotion and no attention has been paid to more complex traits, such as learning and memory [244] with the exception of circadian rhythmicity that was not affected in FRDA flies [245]. We believe these behaviors should also be studied, since they might provide additional information about the protective and anti-degenerative properties of drugs and genetic modifiers.

Besides deeper studies on the physiological phenotypes triggered by frataxin deficiency in the fly, it would be of high relevance to reveal how each cell type or tissue responds to the loss of frataxin. One of the main handicaps in the development of treatments for FRDA is the lack of a cell-specific transcription profile, even when several evidences support specific mitochondrial and cellular alterations upon frataxin silencing in different cells types. Some methodologies have been recently implemented on *Drosophila* to achieve this aim. On one hand, single cell sequencing approaches such as Drop-seq have been successfully applied on *Drosophila* to reveal the cell-specific gene combination in fly embryo and adult midbrain [246,247]. On the other hand, the generation of flies to express the fusion of an *Escherichia coli* DNA adenine methyltransferase to the RNA polymerase II has allowed a targeted DNA adenine methyltransferase identification (DamID) and subsequently enabled genome-wide or tissue/cell specific in vivo profiling of the transcribed genes [248,249]. All these techniques require only a few thousand cells to get robust results and the DamID method has the additional advantage of not needing any cell sorting.

(iii) Finally, up to now, only biased genetic interactions have been performed on the FRDA fly models [46,59,60,61,62]. Unbiased genetic screens should reveal novel pathways involved in the progression and etiology of the disease as well as possible disease biomarkers. Similarly, unbiased pharmacological screens should be conducted to fully exploit the in vivo screening capacity of this organism. An appealing idea might be the possibility to test how the manipulation of different metabolic pathways influences loss of frataxin in the fly. In agreement with this hypothesis, recent studies in isolated platelets from FRDA patients [168] and in a FRDA yeast model [250] have reported a metabolic remodeling. As reviewed by Filadi and colleagues, the mitochondria-ER axis is a pivotal element in such metabolic homeostasis [251].

Both genetic and pharmacological screening approaches should enable the identification of therapeutic compounds that might be relevant not only in the context of FRDA, but also for other mitochondrial diseases.

## Figures and Tables

**Table 1:** Comparison of physiological hallmarks of FRDA in patients and phenotypes observed in FRDA fly models.

Patient Phenotype	Tissue Affected	Genotype	Fly Phenotype
Reduced longevity	Ubiquitous downregulation Ubiquitous downregulation Panneuronal Panneuronal	*Actin*-GAL4>UAS-*fh*IR *Da*-GAL4>*UDIR* *Elav*-GS>*UDIR* *Elav*-GAL4; UAS-Dicer2>*UDIR*	Shortened life span Developmental lethality Shortened life span Shortened life span
Ataxia	Ubiquitous downregulation Panneuronal Larval and adult CNS Serotonergic and Dopaminergic neurons	*Actin*-GAL4>UAS-*fh*IR *Elav*-GAL4; UAS-Dicer2>*UDIR* *c698a*-GAL4>UAS-*fh*IR *Ddc*-GAL4>UAS-*fh*IR	impaired locomotion impaired locomotion No effect No effect
Degeneration and atrophy of DN	mutant photoreceptor neurons	*fh^1^*	Degeneration of neuronal photoreceptors
Degeneration of large sensory neurons from the DRG	PNS PNS	*C96*-GAL4>*UDIR* *Neur*-GAL4>UAS-*fh*IR	Reduced longevity Reduced longevity, impaired locomotion
Degeneration of spinocerebellar tracts	Motorneurons	*D42*-GAL4>*UDIR*	No effect in life span, reduced mitochondrial transport and axonal degeneration
Demyelination of sural nerves and DRG fibres	Panglial	*Repo*-GAL4>*UDIR*	Shortened life span, impaired locomotion, brain degeneration, lipid dyshomeostasis
Hypertrophic cardiomyopathy	Heart	*Hand*-GS>*UDIR*	Heart dilatation an impairment of heart function
Abnormal muscle performance and recovery after exercise	Indirect Flight Muscles	*Mef2*-GAL4>*UDIR*	Reduced ATP production, shortened life span, impaired locomotion

CNS: Central Nervous System; DN: Dentatte nucleus; DRG: Dorsal root ganglia.

## Abbreviations

AAV	adeno-associated virus
Al	aluminium
AMPK	adenosine monophosphate-dependent protein kinase
ATF4	activating transcription factor
BCS	bathocuproine disulphonate
BiP	binding immunoglobulin protein
Cer	ceramide
Cnc	cap-n-collar
CNS	central nervous system
CRISPR	clustered, regularly interspaced, short palindromic repeat
Cu	copper
*CyaY*	*Escherichia coli* frataxin homolog
*Da*	*daughterless*
DamID	targeted DNA adenine methyltransferase identification
DFOB	desferrioxamine B
DFP	deferiprone
dhCer	dihydroceramide
dhSph	dihydrosphingosine
DMT1	divalent metal transporter-1
DN	dentate nucleus
DRG	dorsal root ganglia
DTT	dithiothreitol
eIF2α	α subunit of eukaryotic translation initiation factor 2
EMS	ethyl methanesulfonate
ER	endoplasmic reticulum
ETC	electron transport chain
Fe	iron
Fe–S	iron–sulfur
FFA	free fatty acid
*fh*	*frataxin homolog*
FLP/FRT	flippase and flippase recognition target
FRDA	Friedreich’s ataxia
FXN	human frataxin
GAA	guanine-adenine-adenine
GC/MS	gas chromatography coupled with mass spectrometry
Grx1	glutaredoxine
GSH	reduced glutathione
GSSG	oxidized glutathione
HIFs	hypoxia inducible factors
HRE	hypoxia responsive element
HSAN1	hereditary sensory and autonomic neuropathy type 1
IREs	iron responsive elements
*IRP-1A*	iron regulatory protein 1A
KO	knock-out
lace	palmitoyltransferase
MAMs	mitochondria-associated membranes
*Marf*	mitofusin
MCK	muscle creatine kinase
Mef-2	myocyte enhancer factor-2
Mn	manganese
MPP	mitochondrial processing peptidase
*Mvl*	*Malvolio*
PBA	4-phenylbutyric acid
Pdk1	protein kinase-1
PNS	peripheral nervous system
PPARγ	peroxisome proliferator-activated receptor gamma
RNAi	RNA interference
ROS	reactive oxygen species
SOD1	cytosolic superoxide dismutase
SOD2	mitochondrial superoxide dismutase
Sph	sphingosine
SREBP1	sterol-responsive element-binding protein 1
TPEN	*N*,*N*,*N*′,*N*′-tetrakis(2-pyridinylmethyl)-1,2-ethanediamine
*Tsf*	transferrins
TTM	tetrathiomolybdate
TUDCA	tauroursodeoxycholic acid
UAS	upstream activating sequence
*Yfh1*	yeast frataxin homolog 1
Zn	zinc
